# Development and psychometric validation of a vestibular symptom severity scale

**DOI:** 10.1016/j.bjorl.2026.101885

**Published:** 2026-09-25

**Authors:** Marcia Marcelle Vasconcelos Santos, Leandro Pernambuco, Mariana de Carvalho Leal Gouveia

**Affiliations:** Universidade Federal de Pernambuco (UFPE), Recife, PE, Brazil

**Keywords:** Vestibular balance, Vestibular system, Validation study

## Abstract

**Objective:**

To develop the Self-Perception of Vestibular Symptom Severity Scale (EGV) and to evaluate the validity and reliability of the interpretations derived from its scores.

**Methods:**

This methodological validation study included 100 adults with vestibular symptoms. Instrument development involved an exploratory literature review, item construction through a Delphi panel, and content validation by expert judges. Structural validity was examined separately for the frequency and intensity subscales using exploratory and confirmatory factor analyses. Internal consistency was assessed using Cronbach’s alpha. Test-retest reliability (7- and 14-day intervals) was analyzed using the Intraclass Correlation Coefficient (ICC). Measurement error was estimated through the standard error of measurement and smallest detectable change. Construct validity was evaluated through hypothesis testing by examining associations between EGV scores and validated instruments measuring functional impact, physical activity, autonomy, and attentional awareness.

**Results:**

All items demonstrated satisfactory content validity (coefficients ≥0.80). Exploratory factor analysis supported a unidimensional structure in both subscales (KMO > 0.94; Bartlett’s test p < 0.001), with factor loadings ranging from 0.84 to 0.95. Confirmatory factor analysis indicated adequate model fit. Internal consistency was excellent (α > 0.90). Test-retest reliability showed high stability at 14-days (ICC ≥ 0.80). Higher EGV scores were significantly associated with greater functional limitation, lower physical activity levels, increased dependence, and reduced attentional awareness.

**Conclusion:**

The EGV demonstrated evidence supporting structural validity, reliability, and construct validity, supporting its clinical applicability for measuring vestibular symptom severity. The scale provides a standardized framework for monitoring symptom progression in clinical practice and research.

**Level of Evidence:**

4.

## Highlights

• New patient-reported scale measures severity of vestibular symptoms.
• Instrument quantifies frequency and intensity of vestibular symptoms.
• Validation study shows strong reliability and structural validity.
• Provides standardized monitoring of vestibular symptom severity.

## Introduction

Vestibular symptoms, including vertigo, dizziness, imbalance and motion hypersensitivity result from functional alterations of the vestibular system, which is essential for postural control and spatial orientation. These manifestations may substantially impair daily functioning, leading to reduced mobility, loss of autonomy, emotional distress, and fear of falling. Due to their fluctuating nature, vestibular symptoms often impose a dynamic and unpredictable burden on affected individuals.^1^^,^^2^

Clinical evaluation of vestibular disorders traditionally relies on physical examination and laboratory tests, including videonystagmography and the video Head Impulse Test (vHIT). Although these methods are indispensable for identifying vestibular dysfunction, they do not fully capture patients’ subjective perception of symptoms or their variability over time. For this reason, patient-reported outcome measures are frequently used to complement objective findings and provide a broader understanding of symptom experience.^3^^,^^4^

Self-report instruments offer high ecological validity, reflecting the real-life impact of health conditions more accurately than physiological measures alone.^5^ In individuals with vestibular symptoms, questionnaires are particularly relevant because symptom severity is inherently subjective and may not correspond directly to objective test findings.^6^ Accurate assessment of vestibular symptom severity requires systematic measurement of both frequency and intensity, as greater recurrence and higher intensity are associated with worse functional and emotional outcomes. However, in clinical practice, these parameters are often described qualitatively rather than quantified using standardized criteria.^7^, ^8^, ^9^

Several instruments have been developed to assess disability, functional limitation, and emotional impact related to vestibular disorders.^10^, ^11^, ^12^, ^13^ Nevertheless, most available tools primarily evaluate the consequences of symptoms rather than directly measuring symptom severity itself. Other scales focus on isolated manifestations or specific contexts, providing fragmented information that does not fully represent the multidimensional and fluctuating nature of vestibular symptomatology.^14^

Despite the diversity of assessment instruments, there remains a relevant gap in the absence of scales specifically designed to directly quantify vestibular symptom severity based on core clinical parameters such as frequency and intensity. The lack of a standardized measure compromises consistency in clinical documentation and limits the ability to monitor symptom evolution over time. This limitation may reduce sensitivity to therapeutic changes and hinder longitudinal comparisons in both clinical and research settings.^15^

A structured instrument focused on symptom severity may improve standardization of assessments and enable more reliable monitoring of fluctuations and treatment response. In conditions characterized by variable symptom expression, quantifying frequency and intensity through a unified framework becomes essential for improving clinical decision-making and supporting methodological rigor.

Importantly, the present study does not propose a diagnostic tool. The Self-Perception of Vestibular Symptom Severity Scale (EGV) was developed as a monitoring instrument intended to quantify perceived severity of vestibular symptoms. By systematically measuring frequency and intensity, the EGV aims to provide a standardized and clinically applicable approach to symptom assessment, functioning as a complementary resource to traditional clinical evaluation.

Therefore, this study aims to develop and validate the Self-Perception of Vestibular Symptom Severity Scale (EGV) and to evaluate whether it produces valid and reliable interpretations regarding vestibular symptom severity.

## Methods

### Study design and setting

A methodological validation study was conducted at the Hospital das Clínicas of the Federal University of Pernambuco (HC-UFPE) otoneurology outpatient clinic between September and November 2025.

### Participants

Two groups were involved. For content development and validation, a panel of 14 judges with clinical, academic, or methodological expertise, including representatives of the target population, participated.

For clinical validation, a consecutive sample of 100 adults (22–59 years; mean 45.9 ± 7.14 years) presenting vestibular symptoms was recruited at the outpatient clinic. Most participants were female (77%). The most frequently reported symptoms were dizziness (59%), vertigo (24%), imbalance (9%), and nausea (6%). As participants were recruited from the otorhinolaryngology outpatient clinic while undergoing clinical evaluation, many did not yet have a defined vestibular diagnosis at the time of inclusion. Therefore, eligibility was based on the presence of vestibular symptoms, consistent with the aim of the EGV to assess the self-perceived severity of vestibular symptoms regardless of etiological classification. Individuals with significant neurological impairment affecting comprehension were excluded.

The sample size met COSMIN recommendations for structural validation (minimum 100 participants or at least 7 participants per item).^16^^,^^17^

### Instrument development

The Self-Perception of Vestibular Symptom Severity Scale (EGV) was developed as a Patient-Reported Outcome Measure (PROM) following COSMIN guidelines.

An exploratory literature search was conducted in PubMed/MEDLINE, Scopus, Web of Science, EMBASE, Cochrane Library, SciELO, LILACS, BVS, and Google Scholar to identify existing vestibular symptom instruments and gaps in measurement.

Item construction was performed through a Delphi panel of eight specialists (otolaryngologists, speech-language pathologists, a psychometrician, and target population representatives). Consensus (70%–80%) was achieved after three Delphi rounds.

The final version consisted of two parallel subscales (frequency and intensity), each comprising 12 identical items rated on a 5-point Likert scale (0–4). The scale follows a reflective model, in which all items represent manifestations of a single latent construct: vestibular symptom severity.

The final version of the EGV is presented in Appendix A.

### Content validity

Content validity was assessed by an independent panel of judges using a 5-point Likert scale evaluating relevance, clarity, and representativeness. Individual and total Content Validity Coefficients (CVC) were calculated, adopting ≥ 0.80 as acceptable.

### Structural validity

Structural validity was examined separately for frequency and intensity subscales.

Exploratory factor analysis (principal component extraction with Varimax rotation) was conducted. Sampling adequacy was assessed using the Kaiser-Meyer-Olkin (KMO) index, Bartlett’s test of sphericity, and Measure of Sampling Adequacy (MSA). Factor retention followed Kaiser’s criterion (eigenvalues > 1) and scree plot inspection. Factor loadings ≥0.50 were considered adequate.

Confirmatory Factor Analysis (CFA) was subsequently performed using Diagonally Weighted Least Squares (DWLS), appropriate for ordinal data. Model fit was evaluated using χ²/df, GFI, CFI, TLI, and RMSEA. Acceptable thresholds were χ²/df between 1 and 5, CFI/TLI/GFI ≥ 0.90, and RMSEA ≤ 0.08 (≤ 0.10 considered acceptable for unidimensional ordinal models).

### Internal consistency

Internal consistency was assessed using Cronbach’s alpha. Values ≥0.70 were considered acceptable.

### Reliability and measurement error

Test-retest reliability was evaluated using the Intraclass Correlation Coefficient (ICC), two-way random effects model, ICC.^1^^,^^2^ A minimum ICC ≥ 0.70 was adopted.

Reapplication occurred at 7-days (pilot phase) and 14-days (definitive analysis), using independent subsamples representing 50% of the total sample.

Measurement error was estimated using the Standard Error of Measurement (SEM) and Smallest Detectable Change at 95% confidence (SDC95). Final estimates were derived from the 14-day interval.

### Construct validity

Construct validity was examined through hypothesis testing by analyzing associations between EGV scores and established instruments measuring related constructs, including functional impact (Dizziness Handicap Inventory – DHI), physical activity level (International Physical Activity Questionnaire – IPAQ), functional autonomy (Functional Activities Questionnaire – FAQ), and attentional awareness (Mindful Attention Awareness Scale – MAAS). All instruments were applied using their Brazilian Portuguese versions, previously translated, culturally adapted, and validated for use in Brazil.^18^, ^19^, ^20^, ^21^

Pearson correlation coefficients were calculated to assess the magnitude and direction of associations. Correlations of *r* ≥ 0.50 were considered indicative of convergent validity. When appropriate, group comparisons were performed using independent-samples *t*-tests, and effect sizes were estimated using Cohen’s *d*. Statistical significance was set at p < 0.05.

### Reporting guidelines

This study followed the COSMIN methodology for studies on measurement properties and adhered to the STROBE recommendations for reporting observational research.^22^

### Ethical considerations

The study was approved by the Research Ethics Committee of the Hospital das Clínicas of the Federal University of Pernambuco (HC-UFPE), Brazil (approval no. 7,744,582; CAAE 89740025.1.0000.8807). All participants provided written informed consent prior to data collection.

## Results

The Delphi process demonstrated high agreement among experts, with all items reaching at least 88% consensus. No items were excluded during this stage, and suggested modifications were limited to minor wording adjustments. Content Validity Coefficients (CVCi) ranged from 0.821 to 0.946. Total coefficients per criterion were satisfactory, including relevance (0.875), representativeness (0.861), clarity (0.882), and cultural adequacy (0.872), all exceeding the predefined threshold of 0.80, supporting content validity of the scale (Table 1).

**Table 1:** Content validity coefficients of the EGV.

Criterion	CVC (range)	Total CVC
Relevance	0.821–0.946	0.875
Representativeness	0.839–0.911	0.861
Clarity	0.821–0.946	0.882
Cultural adequacy	0.839–0.929	0.872

Exploratory factor analysis supported a unidimensional structure for both subscales. Sampling adequacy was excellent (KMO = 0.953 for frequency; 0.945 for intensity), and Bartlett’s test of sphericity was significant for both instruments (p < 0.001). Only one factor presented eigenvalue greater than 1 in each subscale. The first factor explained 85.65% of total variance in the frequency subscale and 84.00% in the intensity subscale. Factor loadings ranged from 0.848 to 0.948 for frequency and from 0.847 to 0.950 for intensity, while communalities ranged from 0.749 to 0.904 and from 0.751 to 0.898, respectively (Table 2).

**Table 2:** Exploratory factor analysis summary of the EGV subscales.

Indicator	Frequency	Intensity
KMO	0.953	0.945
Bartlett’s test (p)	<0.001	<0.001
Eigenvalue (Factor 1)	10.278	10.080
Variance explained (%)	85.65%	84.00%
Factor loadings (range)	0.848–0.948	0.847–0.950
Communalities (range)	0.749–0.904	0.751–0.898

Confirmatory factor analysis demonstrated adequate model fit for the proposed unidimensional model in both subscales. The χ²/df ratios were 1.80 (frequency) and 1.88 (intensity). Incremental fit indices were high (CFI ≥ 0.998; TLI ≥ 0.998; GFI ≥ 0.999). RMSEA values were 0.090 for frequency and 0.094 for intensity, indicating acceptable fit considering the ordinal nature of the data and the number of items (Table 3).

**Table 3:** Confirmatory factor analysis fit indices of the EGV.

Fit Index	Frequency	Intensity
χ²/gl	1.8	1.88
GFI	1	0.999
CFI	0.999	0.998
TLI	0.999	0.998
RMSEA	0.09	0.094

Internal consistency was excellent for both subscales. Cronbach’s alpha was 0.985 (95% CI 0.980–0.989) for frequency and 0.983 (95% CI 0.977–0.988) for intensity (Table 4).

**Table 4:** Internal consistency of the EGV subscales.

Subscale	Cronbach’s α	95% CI
Frequency	0.985	0.980–0.989
Intensity	0.983	0.977–0.988

Test-retest reliability assessed at 7-days showed low stability (ICC < 0.50). When reassessed at 14-days, reliability improved substantially. The frequency subscale presented ICC = 0.899 (95% CI 0.824–0.944), and the intensity subscale presented ICC = 0.884 (95% CI 0.780–0.938). Measurement error analysis based on the 14-day interval revealed a Standard Error of Measurement (SEM) of 4.58 for frequency and 4.48 for intensity. The smallest detectable change at the 95% Confidence level (SDC95) was 12.69-points for frequency and 12.43-points for intensity (Table 5).

**Table 5:** Test-retest reliability and measurement error of the EGV (14-day interval).

Subscale	ICC (95% CI)	SEM	SDC95
Frequency	0.899 (0.824–0.944)	4.58	12.69
Intensity	0.884 (0.780–0.938)	4.48	12.43

Construct validity was supported through hypothesis testing. Moderate positive correlations were observed between EGV scores and DHI (frequency: *r* = 0.584; intensity: *r* = 0.571; p < 0.001). Moderate negative correlations were found with IPAQ (frequency: *r* = -0.512; intensity: *r* = -0.523; p < 0.001). Regression analysis indicated that EGV scores explained 25.5% (frequency) and 27.4% (intensity) of the variance in physical activity levels. Significant differences were observed in FAQ scores between low and high severity groups (p < 0.001), with large effect sizes (*d* > 1.0). Negative moderate correlations were also observed between EGV and MAAS (frequency: *r* = -0.547; intensity: *r* = -0.571; p < 0.001), supporting the expected associations (Table 6).

**Table 6:** Construct validity of the EGV through hypothesis testing.

Comparator	Frequency (*r*)	Intensity (*r*)	Additional Analysis
DHI	0.584^a^	0.571^a^	‒
IPAQ	−0.512^a^	−0.523^a^	*R²* = 0.255–0.274
FAQ	‒	‒	*d* = 1.08–1.11^a^
MAAS	−0.547^a^	−0.571^a^	‒

a p < 0.001.

## Discussion

This study developed and validated the Self-Perception of Vestibular Symptom Severity Scale (EGV), demonstrating consistent evidence of content validity, structural validity, reliability, and construct validity. The findings support the use of the EGV as a unidimensional instrument capable of quantifying vestibular symptom severity based on frequency and intensity.

Content validity was supported by high agreement among experts and satisfactory content validity coefficients, indicating that the items adequately represent the construct of vestibular symptom severity. Establishing conceptual clarity at this stage is fundamental to ensure that subsequent psychometric analyses reflect a coherent and theoretically grounded construct.^16^^,^^17^

Exploratory and confirmatory factor analyses supported a unidimensional structure for both subscales. The high proportion of explained variance and strong factor loadings indicate that all items converge toward a single latent construct. Unidimensionality is essential for interpreting a total score as a valid representation of global symptom severity. In the clinical context, this structure simplifies interpretation and facilitates longitudinal monitoring.^23^^,^^24^

Internal consistency was excellent, suggesting strong homogeneity among items. Although very high alpha values may sometimes indicate some degree of overlap among items, their retention was supported by content validity evidence and expert consensus. Furthermore, the high factor loadings and stable confirmatory indices support the coherence of the underlying construct.^23^

Test-retest reliability demonstrated that stability depends on the chosen interval. While the 7-day interval showed low agreement, the 14-day interval resulted in good reliability (ICC ≈ 0.88–0.90). In populations with fluctuating vestibular symptoms, short-term clinical variations may influence stability estimates. The 14-day interval appears to provide a more balanced estimate between recall bias and clinical change.^25^^,^^26^

The estimation of measurement error further strengthens the clinical applicability of the EGV. The smallest detectable change (approximately 13-points) provides a practical threshold for distinguishing real clinical change from measurement variability. This parameter is particularly relevant for rehabilitation and longitudinal follow-up, where treatment decisions depend on meaningful change.^27^^,^^28^

Construct validity was supported by the expected associations with functional impact, physical activity, autonomy, and attentional measures. Moderate correlations with DHI indicate that symptom severity is related to functional handicap, while negative associations with IPAQ and MAAS suggest links between vestibular symptom severity, physical activity, and attentional processes. The large effect sizes observed in functional autonomy comparisons further support the discriminative capacity of the scale. Taken together, these findings provide evidence supporting the construct validity of the EGV and suggest that it reflects clinically relevant aspects of self-perceived vestibular symptom severity.

Clinically, the EGV fills an important gap. Existing instruments often focus on functional impact or emotional consequences rather than directly quantifying symptom severity through frequency and intensity parameters. By separating these two dimensions while maintaining unidimensionality within each subscale, the EGV allows more precise monitoring of symptom progression and therapeutic response.^10^^,^^11^

This study has limitations. Part of the sample was undergoing diagnostic investigation or awaiting treatment, which may have influenced short-term symptom stability. In addition, participants were included based on the presence of vestibular symptoms rather than specific vestibular diagnoses, as many were still undergoing clinical evaluation at the time of recruitment.

Consequently, the applicability of the EGV across specific vestibular diagnostic categories could not be examined in the present study. Test-retest analyses were conducted in independent subsamples, limiting direct comparison between intervals. The excellent reliability observed in the 14-day subsample supports the temporal stability of the EGV over this period. However, because the 7-day and 14-day estimates were obtained from different participants, the higher ICC at 14-days should not be interpreted as evidence of superior reliability relative to the 7-day interval. Responsiveness and anchor-based minimal clinically important difference were not evaluated and should be explored in future studies.

In addition, the sample included only adults aged 22–59 years. Therefore, the generalizability of the present findings to older adult populations remains to be established.

Future research should assess responsiveness, replicate reliability in clinically stable samples, and test the instrument in different etiologies, diagnostic categories, and age groups, including older adults, to enhance generalizability.

Therefore, the EGV demonstrated adequate psychometric properties and provides a clinically applicable tool for measuring vestibular symptom severity in adults. Its structured and standardized approach may contribute to both clinical decision-making and research in vestibular disorders.

## Conclusion

The Self-Perception of Vestibular Symptom Severity Scale (EGV) demonstrated consistent evidence of content validity, unidimensional structure, internal consistency, test-retest reliability, and construct validity. The instrument provides a standardized method for assessing the frequency and intensity of vestibular symptoms in adults and establishes a clinically interpretable threshold for detecting meaningful change over time.

The EGV represents a practical tool for clinical monitoring and research, contributing to a more precise evaluation of vestibular symptom severity across different clinical contexts.

## ORCID ID

Mariana de Carvalho Leal Gouveia: 0000-0003-3358-2791

## Funding

This study was financed in part by the Coordenação de Aperfeiçoamento de Pessoal de Nível Superior – Brasil (CAPES) – Finance Code 001.

## Data availability

The authors declare that all data are available in repository.

## Declaration of competing interest

The authors declare no conflicts of interest.
