# Supplementary material for: Development and psychometric validation of a vestibular symptom severity scale
Source: Braz J Otorhinolaryngol. 2026 Sep 25;92(6):101885. doi: 10.1016/j.bjorl.2026.101885 (PMC13635710; doi:10.1016/j.bjorl.2026.101885)
Supplement: Supplementary file 1 [file mmc1.docx]

BJORL-D-26-00180_Supplementary Material

**Appendix TA**

| **Self-Perception of Vestibular Symptom Severity Scale (EGV)** | | | | | | | | | | |
| --- | --- | --- | --- | --- | --- | --- | --- | --- | --- | --- |
| *Original Brazilian Portuguese Version* | | | | | | | | | | |
| **Instruções** | | | | | | | | | | |
| Nos últimos 7-dias, como você percebeu o seu sintoma nas situações abaixo? | | | | | | | | | | |
| Você encontrará 12 situações em que o seu sintoma pode aparecer ou piorar. | | | | | | | | | | |
| Para cada situação, responda duas vezes: | | | | | | | | | | |
| • Uma vez para a frequência do sintoma | | | | | | | | | | |
| • Outra vez para a intensidade do sintoma | | | | | | | | | | |
|  | | | | | | | | | | |
| **Marque um número para Frequência e um número para Intensidade em cada item** | | | | | | | | | | |
| Escala de resposta – Frequência | | | | | | | | | | |
| 0 – Nunca | | | | | | | | | | |
| 1 – Raramente (quase nunca) | | | | | | | | | | |
| 2 – Às vezes (apareceu algumas vezes) | | | | | | | | | | |
| 3 – Frequentemente (apareceu muitas vezes) | | | | | | | | | | |
| 4 – Quase sempre (presente quase todos os dias) | | | | | | | | | | |
|  | | | | | | | | | | |
| Escala de resposta – Intensidade | | | | | | | | | | |
| 0 – Não senti nada | | | | | | | | | | |
| 1 – Incômodo leve (pouco incômodo) | | | | | | | | | | |
| 2 – Incômodo moderado (incômodo médio) | | | | | | | | | | |
| 3 – Incômodo forte (incômodo intenso) | | | | | | | | | | |
| 4 – Incômodo muito forte (muito intenso e difícil de suportar) | | | | | | | | | | |
|  | | | | | | | | | | |
| **Pergunta** | **Frequência** | | | | | **Intensidade** | | | | |
|  | **0** | **1** | **2** | **3** | **4** | **0** | **1** | **2** | **3** | **4** |
| 1. Seu sintoma apareceu ou dificultou seu equilíbrio ao ficar em pé ou andar? | ○ | ○ | ○ | ○ | ○ | ○ | ○ | ○ | ○ | ○ |
| 2. Sentiu medo ou insegurança de cair, ou caiu ao caminhar ou ao realizar tarefas em pé? | ○ | ○ | ○ | ○ | ○ | ○ | ○ | ○ | ○ | ○ |
| 3. Sentiu que poderia se desequilibrar ao subir ou descer escadas? | ○ | ○ | ○ | ○ | ○ | ○ | ○ | ○ | ○ | ○ |
| 4. Ao se levantar da cama ou cadeira, seu sintoma apareceu ou piorou? | ○ | ○ | ○ | ○ | ○ | ○ | ○ | ○ | ○ | ○ |
| 5. Ao se deitar ou virar a cabeça, sentiu que o ambiente girava ou balançava? | ○ | ○ | ○ | ○ | ○ | ○ | ○ | ○ | ○ | ○ |
| 6. O sintoma apareceu ou piorou em lugares com muita gente ou movimento? | ○ | ○ | ○ | ○ | ○ | ○ | ○ | ○ | ○ | ○ |
| 7. Sentiu desconforto com luzes, brilhos, cheiros, barulhos ou telas? | ○ | ○ | ○ | ○ | ○ | ○ | ○ | ○ | ○ | ○ |
| 8. Percebeu enjoo, palidez ou suor frio junto com o sintoma? | ○ | ○ | ○ | ○ | ○ | ○ | ○ | ○ | ○ | ○ |
| 9. Sentiu zumbido ou sensação de ouvido tampado? | ○ | ○ | ○ | ○ | ○ | ○ | ○ | ○ | ○ | ○ |
| 10. Parou de fazer tarefas do dia a dia por causa do sintoma? | ○ | ○ | ○ | ○ | ○ | ○ | ○ | ○ | ○ | ○ |
| 11. Evitou movimentos com medo do sintoma reaparecer? | ○ | ○ | ○ | ○ | ○ | ○ | ○ | ○ | ○ | ○ |
| 12. O sintoma causou desânimo, frustração ou ansiedade? | ○ | ○ | ○ | ○ | ○ | ○ | ○ | ○ | ○ | ○ |
